# Presenting the improved possibility for staying well might be better than talking about change in risk: Use of the Non-Occurrence Probability Increase (NOPI)

**DOI:** 10.3109/02813432.2013.811951

**Published:** 2013-09

**Authors:** Bertil Hagström, Ronny K. Gunnarsson, Mark Rosenfeld

**Affiliations:** Research and Development Unit, Primary Health Care and Dental Care, Southern Älvsborg County, Region Västra Götaland, Sweden

**Keywords:** Cardiovascular diseases, drug therapy, general practice, patient compliance, patient education, probability, risk assessment, Sweden

## Abstract

**Objective:**

Talking about risk with patients is problematic since the individual's risk is not addressed and is usually very low. This study aimed to see how fact presentation influenced the decision-making process for general practitioners concerning treatment for the prevention of cardiovascular disease. Rather than looking at the risk of becoming ill, often presented as high figures of relative risk reduction (RRR), it could be useful to present the probability of staying well, i.e. from the concept of non-occurrence probability (NOP) and non-occurrence probability increase (NOPI) – simply a single measure of change in NOP.

**Design:**

General practitioners (GPs) had personal response keypads to answer two questions, presented differently, concerning whether they would allow themselves to be treated or not be treated for the risk of cardiovascular death.

**Setting and subjects:**

Five audiences consisting of general practitioners attending lectures.

**Results:**

When the question was presented as RRR, 68% and 86%, respectively, of the physicians responded that they would take the decision to treat. When presented as the concept of NOPI the figures were reduced to 18% and 16%, respectively (p < 10–6).

**Implications:**

Developing tools to explain treatment effect is crucial to enhancing health care quality. Since NOPI is one potential way of presenting prevention of risk we encourage future research to evaluate the NOPI concept compared with RRR and absolute risk reduction (ARR).

- Developing tools to explain treatment effect is crucial to enhancing health care quality.
- The Non-Occurrence Probability Increase (NOPI) provides patients with a more realistic view of how treatment alters the probability of staying well than the RRR, which presents the lowered risk of becoming ill. RRR and NOPI may be considered highly divergent in the presentation of treatment effects and strongly affect physician decision-making concerning medication.

## Introduction

Talking about risk with patients is problematic since the individual's risk is not addressed. Furthermore, words influence thought and behaviour and the use of the word “risk” may cause worry [1,2]. Understandable ways of discussing risk are essential. This short communication introduces two acronyms, NOP (non-occurrence probability) and NOPI (non-occurrence probability increase), whereby it is possible to avoid the concept of risk.

If a treatment gives a relative risk reduction (RRR) for a major cardiovascular event by 50% in a population the benefit (to society) seems obvious even though absolute risk reduction (ARR) is low. Presenting a question differently, such as both by RRR and as ARR, has a considerable effect on the intended use of a treatment and RRR seems more persuasive [3,4]. However, it is uncertain whether presenting RRR is likely to help patients make decisions about medication more consistent with their own values. ARR is supposedly more interesting than RRR. Whether a drug reduces mortality from 2% to 1% or from 40% to 20% is essential.

Another perspective is NOP, which makes the magnitude of “the risk” more understandable [5,6]. The lack of research is considerable [3]. This report aimed to investigate how another perspective on treatment effect and presentation influences the physician's attitude.

## Material and methods

BH gave five lectures to primary care physicians (GPs) on medication for risk reduction in Sweden from September 2007 to March 2009 to audiences of 145 persons who were representative of Swedish GPs. Before discussing the risk concept two examples of treatment effects were presented: first as RRR and shortly after as the non-occurrence probability increase NOPI – a single measure of NOP change.

The two examples were first presented as RRR without baseline probability: “Would you reduce your risk for cardiovascular death by 50% during a 10-year period with medication?” [7], and “Would you reduce your risk by 33% for a cardiovascular event during a 3–4 year period with medication?” [8]. Then as NOPI: “Would you increase your chance of avoiding a cardiovascular death during a 10-year period from 98% to 99% with medication?” [7], and “Would you increase your chance of avoiding a cardiovascular event during a 3–4 year period from 97% to 98% with medication?” [8]. The audience responses to the questions were recorded using an audience response system of personal response keypads and a response receiver (Turning point^®^ by Turning Technologies).

## Results

When the first example was presented as RRR 68% of the primary care physicians agreed (90/132), and when presented as NOPI only 18% agreed (25/136). When the second example was presented as RRR 86% agreed (55/64), and when presented as NOPI 16% agreed (10/62) (Table I). The difference between RRR and NOPI was of similar magnitude regardless of whether the example described an uncomplicated (example 1) or a high-risk patient (example 2).

**Table I.** General practitioners’ perception of the difference between risk and chance.

	Treatment effect presented as relative risk reduction (RRR)	Treatment effect presented as non-occurrence probability increase (NOPI)	P-value (chi-squared with Yates's correction)
Example 1: You are a 55-year-old woman. Drugs may affect probability of future cardiovascular death^a^	Would you reduce your risk for a cardiovascular death during a 10-year period by 50% with medication? 68% (90/132) of GPs said yes	Would you increase your chance of avoiding a cardiovascular death during a 10-year period from 98% to 99% with medication? 18% (25/136) of GPs said yes	< 0.001
Example 2: You are a 60-year-old man and have at least two risk factors – smoking, stroke, transient ischaemic attack (TIA), or hereditary – for cardiovascular disease Drugs may affect the probability of a future cardiovascular event?^b^	Would you reduce your risk for a cardiovascular event during a 3–4 year period by 33% with medication? 86% (55/64) of GPs said yes	Would you increase your chance of avoiding a cardiovascular event during a 3–4 year period from 97% to 98% with medication? 16% (10/62) of GPs said yes	< 0.001

Notes: ^a^Wilhelmsen et al. [7]. ^b^Sever et al. [8].

## Discussion

In this study we compared the agreement to treatment options for cardiovascular preventive medication presented either as RRR or as NOPI to a group of Swedish GPs. We found that few physicians accepted pharmacological treatment when the effect was presented as NOPI while most would accept treatment when the effect was presented as RRR. Physicians perceived the options completely inversely depending on how treatment effect was presented [9].

There are methodological weaknesses in an audience-response system. However, it was estimated that more than 90% responded to each question. The second question was added to the lectures later than the first and therefore had fewer respondents, and was also presented after the first and could therefore have introduced a systematic error. A better approach might have been to let GPs answer only the examples presented with either RRR or NOPI, not both. However, it seems unlikely that this potential error would explain all the difference between RRR and NOPI option responses. One can argue that conceptual confusion may arise when comparing RRR with NOPI. Both RRR and ARR deal with the lowered risk of becoming ill while NOPI deals with the increased chance of staying well. Thus, RRR and NOPI are extreme opposites in how treatment effect is presented.

Risk evaluation is complex and the word risk might be frightening to some patients [10,11]. Talking about increased probability of staying healthy may therefore be a better option. Yet, a NOP increase from 98% to 99% giving a NOPI of 1% is not as convincing an argument as a 50% relative risk reduction for cardiovascular death [12]. Not even the GPs in this study chose treatment when NOPI was small.

Developing tools to explain treatment effect is crucial to enhancing health care quality [13–16]. Since NOPI is one potential way of presenting prevention of risk we encourage future research to evaluate the NOPI concept compared with RRR and ARR.
